# Phase IV study on the use of benzydamine mouthwash in radiation-induced oral mucositis in patients with head and neck cancer

**DOI:** 10.3389/fonc.2024.1345129

**Published:** 2024-02-26

**Authors:** Paolo Bossi, Valeria Tellone, Giorgio Di Loreto, Sara Fioravanti, Enrica Salvatori, Alessandro Comandini

**Affiliations:** ^1^ Department of Biomedical Sciences, Humanitas University, Milan, Italy; ^2^ Medical Oncology and Hematology Unit, Humanitas Cancer Center, IRCCS Humanitas Research Hospital, Milan, Italy; ^3^ Global Medical Department, Angelini Pharma S.p.A., Rome, Italy; ^4^ Pharmacometrics & Clinical Supply, Angelini Pharma S.p.A., Rome, Italy

**Keywords:** head and neck cancer (HNC), benzydamine, oral mucositis (OM), prevention, treatment

## Abstract

**Introduction:**

Oral mucositis (OM) is a main side effect of treatment for head and neck cancer (HNC) and causes severe pain, reduces quality of life, and may interrupt HNC treatment. This study assessed the activity and feasibility of benzydamine mouthwash in the prevention and treatment of radiation-induced OM in patients with HNC during radiation therapy (RT).

**Methods:**

This phase IV, international, open-label, single-group study conducted from December 2021 to September 2022. In total, 89 patients were enrolled across seven centers in Hungary and Poland. Patients used benzydamine mouthwash at home two to three times daily. Data were collected during clinical visits at baseline (V0, start of RT) and then weekly for seven visits (V1–V7). The safety population and the modified intention-to-treat (m-ITT) analysis sets contained 89 patients; the per protocol (PP) analysis set contained 67 patients.

**Results:**

The m-ITT set was 80.9% male; mean age was 61.4 years. At baseline, 73.0% of patients had stage T3-T4, 23.6% had stage T1-T2, 61.8% had stage N2-N3, and 34.9% had stage N0-N1. Within the m-ITT population, 33.7% (n=30) responded to treatment (NRS < 5) during the study. The PP set responded similarly (29.9%). Most patients were treatment compliant (n=77; 86.5%). OM severity was assessed using the WHO OM grading scale. No patients had severe mucositis at baseline or V1. At V7, 34.1% had mild mucositis, 45.1% had moderate mucositis, 15.9% had severe mucositis, and 1.2% had life-threatening mucositis. In total, 26 patients (29.2%) developed severe mucositis during the study period (V2–V7). From V1 to V4, one patient reported hospitalization due to mucositis or associated complications, two patients at V5, three patients at V6, and four patients at 7.

**Discussion:**

This was the first study to assess feasibility of a treatment for radiation-induced OM with benzydamine mouthwash in patients with HNC. Treatment compliance suggested that benzydamine was well tolerated in patients with moderate to severe mucositis. Benzydamine’s anesthetic and anti-inflammatory properties might have reduced pain, which potentially influenced patients’ compliance with RT. Few patients in the study required hospitalization for OM or an associated complication, suggesting that benzydamine might improve healthcare resource utilization.

## 1 Introduction

Head and neck cancer (HNC) accounts for approximately 550,000 cases worldwide per year (1, 2) and includes malignant tumors that most commonly arise from the moist squamous cell mucosa or lining of the head and neck regions. Treatments for managing HNC include surgery, radiation therapy (RT) and systemic therapies. Indeed, a multidisciplinary treatment schedule should be established in all cases, both to improve survival rates and to treat adverse events (AEs) (3, 4). Of all the AEs, mucositis poses a significant challenge for both the patients and the physicians, being present in virtually all patients with HNC who receive radiotherapy with or without systemic therapies (5).

Oral mucositis (OM) refers to mucosal damage secondary to cancer therapy and occurs in approximately 20% to 40% of patients receiving conventional chemotherapy (CT), 80% of patients receiving high-dose CT, and nearly all patients receiving RT on head and neck mucosa (6). OM is typically very painful and may become severe enough to prevent patients from speaking, eating, drinking, or swallowing (7). Mucositis-associated pain is the most frequently reported symptom, although oral discomfort persists in almost a half of patients for extended periods of time, even following resolution of visible OM (8). OM symptoms and its consequences—such as dehydration, weight loss, and systemic infections (9)—impair nutritional intake, reduce compliance to oncology treatment and the treatment’s efficacy, and adversely impact economic outcomes, especially as a result of increased hospitalization and the need for parenteral or tube feeding (6, 10–13).

Because of the different mechanisms underlying OM pathogenesis, many interventions have been tested. Nevertheless, there is no clearly effective therapy for OM, and strategies for its management include preventive measures and therapeutic approaches, such as improved oral hygiene and oral care (to eliminate any irritants from the oral mucosa and reduce the risk of superinfections), systemic analgesics, topical palliative agents (e.g., MuGard), combined treatments (e.g., lidocaine solutions, “magic mouthwash” preparations), or antioxidants (5). Moreover, anti-inflammatory agents may also be suggested (14).

Benzydamine is a drug with a long history of safe use in patients and shows local anesthetic and analgesic properties and anti-inflammatory activity. Its elective therapeutic use is the topical control of acute inflammation and pain. The main mechanism is the drug’s inhibition of cytokine production, which results in decreased vessel permeability and inhibition of leukocyte migration and degranulation at the inflammation site. Benzydamine has been demonstrated to inhibit the production of proinflammatory cytokines such as tumor necrosis factor-α and interleukin-1β (15–17). In addition, benzydamine has been studied for its antimicrobial activity using different techniques against more than 100 bacterial strains belonging to different species, comprising bacteria, yeasts, and fungi (18).

Inflammation is considered to be an important tissue reaction in radiotherapy and CT-induced OM (19). The 2014 MASCC/ISOO guidelines (6) recommended the use of benzydamine mouthwash for the prevention of OM in patients with HNC who are receiving moderate-dose RT (up to 50 Gy) without concomitant CT. The 2020 MASCC/ISOO guidelines confirm the previous recommendation with new evidence (14). Moreover, the 2020 guidelines recommend the use of benzydamine mouthwash for the prevention of OM in patients with HNC who are receiving RT and CT, without RT dose specification (14).

Because data on the use of benzydamine throughout the course of RT is lacking, we aimed to provide evidence on the feasibility of a preventative/therapeutic approach of radiation-induced OM with benzydamine mouthwash in patients with HNC. Moreover, data on OM pain control during RT, percentage of severe mucositis (grades 3/4 of the WHO), duration and time to onset of severe mucositis, nutritional status of HNC patients, compliance to oncology therapy and to benzydamine treatment, healthcare resources consumption, opioid analgesics’ use, and safety data were collected.

## 2 Materials and methods

### 2.1 Study design

This was a phase IV, international, open label, single-group study conducted from December 2021 to September 2022. The objective was to assess the activity and feasibility of benzydamine mouthwash in the prevention and treatment of radiation-induced OM in patients with HNC from first day of RT through the end of RT.

A total of seven centers were involved across two countries: Hungary and Poland. Before study start-up, all relevant regulatory authorities and ethics committees provided review and written approval for all clinical study documentation (protocol 030(Z)WO19247) and adequacy of investigational sites. The study was conducted in accordance with the Declaration of Helsinki, Good Clinical Practice principles, and all applicable regulatory requirements including ICH E6 Good Clinical Practice (R2). All relevant regulatory and ethical committees provided review and written approval for all clinical study documentation and investigational sites.

Before enrolment into the study, patients were fully informed about the purposes of the research, as well as all procedures relating to their involvement. Before entering the study, a written and signed informed consent form and the Declaration of Consent for Processing of Personal Data were obtained from all patients.

### 2.2 Study treatment and study outcomes

Benzydamine hydrochloride 1.5 mg/mL mouthwash was assigned to the patients for the prevention/treatment of radiation-induced OM, according to the investigator’s recommendations and to the relevant local SmPC (Summary of Product Characteristics).

Treatment of 15 mL (1 tablespoon) of concentrated or diluted (with water) solution was taken at home, two or three times a day, but not more than five times a day. Patients were asked to wash the mouth and throat for 20 to 30 seconds, according to the investigator’s indications and the local product’s SmPC.

Treatment compliance was defined as taking ≥ 80% of the total dose of benzydamine treatment assigned by the investigator.

The primary outcome for this study was the number of responders, defined as the number of patients with HNC who had OM pain intensity <5 (numeric rating scale [NRS]), expressed as a percentage, at visits 0 to 7/early treatment termination visit (ETTV).

The key secondary outcomes were the number of compliant patients, change in score on the WHO OM grading scale from Visit 0 through Visit 7/ETTV, number of days’ duration and time to onset of severe mucositis, percentage change in body weight and need for nutritional support, number of days duration of RT/CT administered as well as discontinuation, dose modifications and delays, number of days of hospitalization, number and type of opioid analgesics used for OM pain, and changes from Visit 0 in vital signs, physical examination and adverse events to Visit 7/ETTV.

### 2.3 Study procedures

Data were collected during clinical visits at baseline (V0, start of RT), Visit 1 (Day 7 ± 1), Visit 2 (Day 14 ± 1), Visit 3 (Day 21 ± 1), Visit 4 (Day 28 ± 1), Visit 5 (Day 35 ± 1), Visit 6 (Day 42 ± 1) during RT, and Visit 7 (Day 49 ± 1, Final visit/End of RT).

At baseline, which was defined as the start of RT, all patients were fully informed by the investigator about the purpose of the study, patients received standard study information, and patients provided written informed consent forms. Key study data including demographics, medical history, oncology history, physical examination, vital signs, current oncology status and treatment, Eastern Cooperative Oncology Group (ECOG) status, OM evaluation, benzydamine treatment prescription, and AEs were collected.

Eligible patients were enrolled in the study, and one visit per week during the radiotherapy period was scheduled. During these visits, data were collected and recorded in source documents by the investigator who then reported into the electronic case report form. Patients were asked to self-report data on OM pain assessment (NRS) on paper during each visit. Patients were instructed to consider any pain due to mucositis in oral cavity and asked to select the number (0/not present -10/pain as bad you can imagine) best describing the intensity of pain over the past 7 days. OM severity was evaluated at each visit by physicians having at least a 2-year experience in HNC treatment. Sites were trained on OM severity scoring via online training using the WHO classification scale.

### 2.4 Patient population

The study population consisted of male and female patients 18 years or older with a histologic or cytologic diagnosis of stage III or IV HNC in subsites—including oral cavity, oropharynx, larynx and hypopharynx, according to the VIII American Joint Committee on Cancer (AJCC) staging system—who were candidates and were about to start RT. Exclusion criteria included reported allergy to benzydamine or related components of the formulation used; prior head and neck RT; having received palliative treatment; any metastatic disease, cognitive impairment, or significant comorbid conditions; OM due to other conditions; use of other oromucosal products, rinses, or anti-inflammatory mouthwash solutions; use of antifungal or antibiotic drugs; or having received other therapies that cause mucositis.

### 2.5 Statistical analysis

Because of the nature of the single-arm study, all statistical analysis was descriptive. Continuous variables were described by mean, standard deviation (SD), median, interquartile range (IQR), minimum, maximum, and number of available patients. Categorical variables were described by frequency and percentages. Two-sided 95% confidence intervals (CIs) were provided for the primary and secondary outcome variables. When relevant, 95% CIs were provided for demographic variables as well. No comparison test was conducted. SAS version 9.4 was used for analysis.

This Phase IV single-arm study had no hypothesis to test, and sample-size justification was based on precision expected for the primary outcome (e.g., the number of responders). A target sample size of 100 patients (including an assumption of 20% dropouts) was considered appropriate to evaluate the percentage of responders, defined as the number of patients with HNC who had OM pain intensity < 5 (NRS).

The following analysis populations were defined for statistical analysis:

- The per-protocol (PP) population: defined as all the patients with treatment compliance to the study medication ≥ 80% and all NRS evaluations, from first day of RT through 4 weeks, with no major protocol violations.
- The modified intention-to-treat (m-ITT) population: defined as all the patients who took at least one dose of the study medication and had one NRS evaluation at Visit 0 and one NRS evaluation post-Visit 0. The last-observation-carried-forward method was implemented as imputation scheme to handle missing data.
- The safety population (SP): defined as all the patients who took at least one dose of the study medication.

## 3 Results

### 3.1 Study patients

**Figure 1:**
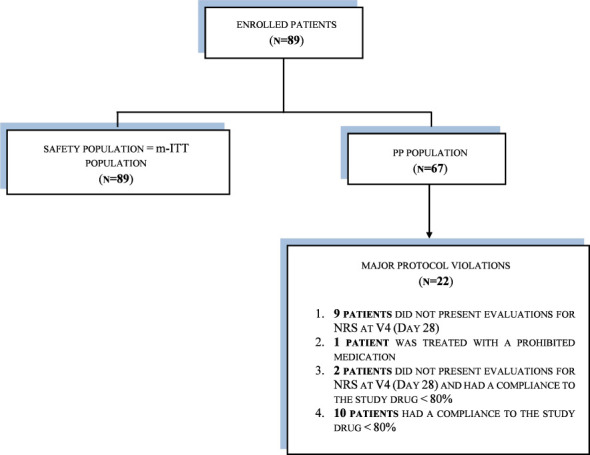
Disposition of patients.

It was planned that a sample of 100 patients (including an assumption of 20% dropouts) would be included in the study; 89 were actually enrolled into the study from seven sites in two European countries: Poland and Hungary. Three analysis sets were used: the SP and m-ITT analysis sets each contained 89 patients; the PP analysis set contained 67 patients, because 22 patients had a major protocol violation and were excluded from the PP analysis population (see **Figure 1**). As the patients in the SP and m-ITT analysis populations were exactly the same, all results are presented as m-ITT. As shown in **Figure 1**, there were 89 patients in the m-ITT sett and 67 patients in the PP set.

In the m-ITT set, the majority of the study population was male (80.9%) and their mean age was 61.4 years (range: 41-77 years). All patients were White (**Table 1**).

**Table 1:** Patient demographics.

	m-ITT	PP
Age (years)		
N	89	67
Mean (SD)	61.36 (8.37)	61.97 (8.49)
95% CI	59.60; 63.12	59.90; 64.04
Q1-Q3	56.00 - 67.00	57.00 - 68.00
Median	61.00	62.00
Min-Max	41.00 - 77.00	41.00 - 77.00
Gender		
M	72 (80.9%)	54 (80.6%)
F	17 (19.1%)	13 (19.4%)
Race		
White	89 (100.0%)	67 (100.0%)

Among the m-ITT set at baseline, 88 patients (98.9%) had head and neck cancer stage III or IV; one patient had missing information (**Table 2**). The disease subsite was the oral cavity for 33.7% of patients, the oropharynx and hypopharynx for 36.0% of patients, and the larynx for 29.2% of patients. A majority of patients had stage T3-T4 (73.0%), nearly a quarter (23.6%) had stage T1-T2. 61.8% had stage N2-N3, and approximately a third (34.9%) had stage N0-N1.

**Table 2:** Disease characteristics, m-ITT.

Characteristic	m-ITT (%)
HNC stage III or stage IV	
Missing information	1 (1.1%)
Disease subsite	
• oral cavity	33.7%
• oropharynx and hypopharynx	36.0%
• larynx	29.2%
• missing information	1.1%
T stage	
• T1-T2	23.6%
• T3-T4	73.0%
• TX	1.1%
• T0missing information	1.1%
N stage	
• N0-N1	34.9%
• N2-N3	61.8%
• NX	2.2%
• Missing information	1.1%

### 3.2 Treatment response

The activity of benzydamine mouthwash in the prevention/treatment of radiation-induced OM in patients with HNC, from the first day of RT through end of RT/ETTV was assessed in all patients in the study. The activity was assessed by the primary study outcome, which was response to the benzydamine mouthwash, operationally defined as the number of patients with HNC who had OM pain intensity < 5 (NRS), expressed as a percentage, at Visits 0 to 7/ETTV. Of the 89 patients in the m-ITT set, approximately a third (n=30, 33.7%) were responders (**Table 3**). The level of response in the PP was similar (29.9%).

**Table 3:** The number of HNC patients with OM pain intensity < 5 (NRS) until Visit 7.

Patient type	m-ITT (N=89)	PP (N=67)
Responder	30 (33.7%)	20 (29.9%)
Nonresponder	59 (66.3%)	47 (70.1%)

The primary study outcome was also investigated in an explorative way by performing the primary analysis using data from Visit 0 to Visit 4 only. Compared with the corresponding results from Visits 0 to Visit 7/ETTV (**Table 3**), the exploratory primary analyses revealed a higher number of responders during Visits 0 to Visit 4, 52.8% in the m-ITT set and 52.2% in the PP set (**Table 4**).

**Table 4:** The number of HNC patients with OM pain intensity < 5 (NRS) until Visit 4.

Patient type	m-ITT (N=89)	PP (N=67)
Responders	47 (52.8%)	35 (52.2%)
Non responders	42 (47.2%)	32 (47.8%)

### 3.3 Treatment compliance

Of the 89 patients in the m-ITT set, the vast majority were found to be treatment compliant (n=77, 86.5%) (**Table 5**). Within the m-ITT, the mean total extent of exposure was 41.9 days, with a minimum of 14 days to a maximum of 65 days. Within the PP, the mean total extent of exposure was similar: 44.7 days (data not shown).

**Table 5:** Number of compliant patients from Visit 0 to 7/ETTV.

Compliance	m-ITT (N=89)	PP (N=67)
<80%	12 (13.5%)	
≥80%	77 (86.5%)	67 (100.0%)

In total, 135 treatment-emergent adverse events (TEAEs) were reported during the study; of these, only two were adverse drug reactions (ADRs), and they occurred in separate patients. The vast majority of TEAEs (93.3%) were reported as unlikely to be correlated to the investigational medicinal product. In total, four patients were reported to have at least one SAE. One patient was reported to have an “infection and infestation” (neutropenic sepsis), another patient was reported to have a “metabolism and nutrition disorder” (Cachexia) and a “general disorder and administration site condition” (pyrexia), one patient was reported to have a “nervous system disorder” (Syncope), and one patient was reported to have an “infection and infestation” (COVID-19) (data not shown).

### 3.4 Severity of OM and NRS response

**Figure 2:**
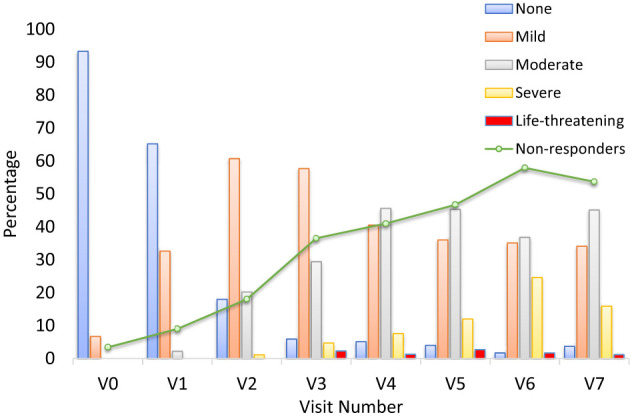
Grade of oral mucositis by visit and number of patients with NRS ≥5 (non-responders) (m-ITT) at V0-V7. The bar graph presents the percentage of patients at each visit who had oral mucositis, separated by severity. Severity was assessed using the World Health Organization (WHO) oral mucositis grading scale. The investigator provided a score corresponding to the grade of mucositis of the patient: 0 = None, 1 = Mild, 2 = Moderate, 3 = Severe, 4 = Life-threatening. The line represents the non-responder patients with an NRS value ≥5.

Severity of OM was assessed through change in score on the WHO OM grading scale from Visit 0 to Visit 7. According to the WHO classification, severity of OM is graded on the following scale: 0 (none), 1 (mild, oral soreness and erythema), 2 (moderate (erythema, ulcers, solid diet tolerated), 3 (severe, oral ulcers, liquid diet only), or 4 (life-threatening, oral alimentation impossible). Nearly all patients (93.3%) had no mucositis at Visit 0 (**Figure 2**). By contrast, at Visit 7, very few patients (3.7%) did not have mucositis; approximately a third (34.1%) had mild mucositis, nearly half (45.1%) had moderate mucositis, 15.9% had severe mucositis, and 1.2% had life-threatening mucositis.

**Figure 3:**
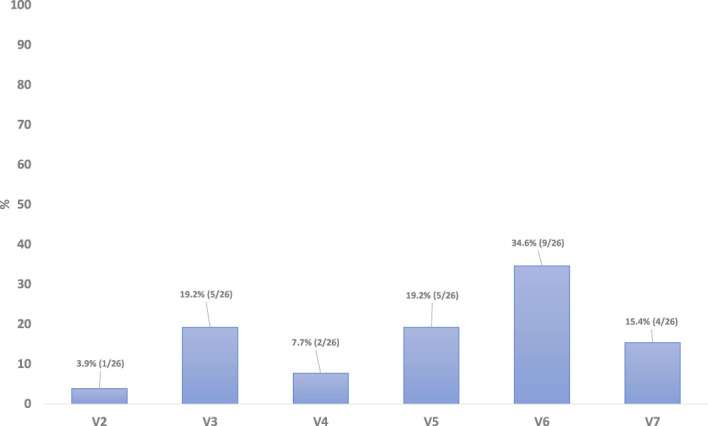
Duaration and time to onset of severe OM, from first day of RT through end of RT/ETTV, m-ITT.

Duration and time to onset of severe OM was also assessed during the RT. There were no reports of patients with severe mucositis (WHO OM grade 3 or 4) at baseline (Visit 0) or Visit 1 (**Figure 3**). In total, 26 patients (29.2% of the m-ITT set) were reported to have developed severe mucositis (WHO OM grade 3 or 4) during the study period, across Visits 2 through 7 inclusive. In this graph it is evident that the number of non-responder patients increases during the study, as expected in parallel with the worsening of OM.

Nutritional support (i.e., need of a feeding tube) and type of enteral support (i.e., partial or total and number of days of need) was assessed at Visits 0 to 7/ETTV. In the m-ITT, at Visit 1, a majority of patients did not need nutritional support for OM (n=70, 78.7%). At Visit 7, a majority of patients still did not require nutritional support (58.4%), lower than at Visit 1 (data not shown).

In addition, healthcare resource use consumption was assessed by the number of days of hospitalization on which the main reason was mucositis or an associated complication from Visit 0 to Visit 7. In the m-ITT set, at the first four visits, only one patient reported hospitalization due to mucositis or associated complications; only two patients had this outcome at Visit 5, three patients at Visit 6, and four patients at Visit 7 (data not shown).

Furthermore, the use of opioid analgesics for OM pain was also assessed and measured by the number and type of opioid analgesics used for OM pain at visits 0 to 7. In the m-ITT, opioid analgesics were used for OM pain among 47 (52.8%) patients.

## 4 Discussion

In this Phase IV study, we assessed the feasibility and activity of benzydamine mouthwash on radiation-induced OM in patients with HNC treated with RT. The high compliance rate (86.5%) observed in the study and the long duration of benzydamine administration (mean exposure about 6 weeks) confirm the feasibility of the treatment throughout the RT. The results demonstrate that the use of benzydamine was also safe: very limited adverse events were reported that were linked to the investigational product (two ADRs).

In this study, approximately a third (33.7%) of all enrolled patients had an OM pain intensity score of <5 at study end, indicating that they were responders to treatment during the treatment period. In addition, only 26 of the 89 (29%) patients developed severe OM during the study period, suggesting that benzydamine mouthwash treatment may have contributed to preventing severe OM in the majority of patients. This finding is relevant because of the prevalence of severe OM reported in the literature, where it is reported that around 60% to 80% of patients with HNC who receive RT develop severe OM (20).

Moreover, the study showed a relatively low use of opioids (53%) compared with the literature. It is commonly reported in the literature that the majority of patients with OM and OM-related pain are administered opioids, and figures range from 78% to 97%. A review of the medical records of 165 patients with HNC demonstrated that more than 80% of patients required opioids (21); similarly, Alfieri et al. (22) reported that 97% of patients in their study received opioids, and Söderlund Schaller et al. (23) reported opioid use by 78% of patients. This could support the analgesic effect of benzydamine in this setting of treatment, even though with the limitation of indirect comparison.

The finding that almost none of the patients in the current study required hospitalization for OM or an associated complication suggests that benzydamine treatment may have had a beneficial impact on healthcare resource use, particularly compared with prevalence of hospitalization due to mucositis reported in the wider literature (24). A review showed that of the 450 patients included, 33% of patients with severe OM were hospitalized, 16% of those with moderate OM were hospitalized, and 21% of those with mild OM were hospitalized (25). Figures are similar across the literature (26, 27),

These collective findings showed that benzydamine mouthwash was well tolerated and may reduce the intensity of symptoms of OM in patients with OM.

Our study presents some limitations. First, because of the single-arm design, there was not a comparator cohort, and analyses were descriptive and did not include hypotheses testing. Also, it was not possible to gather all study data at all assessment windows, and therefore data are missing for some study variables at some study visits. Despite efforts to collect data at all data points, data quality was dependent on the data documentation available in the medical records.

However, this study was based on a prospective collection of data in patients with OM, which is not a commonly reported within this patient population, according to the existing literature.

In conclusion, this was the first prospective study to assess the feasibility of a preventative/therapeutic approach of radiation-induced OM with benzydamine mouthwash in patients with HNC. The majority of enrolled patients were found to be treatment compliant (86.5%), suggesting that benzydamine was well tolerated even in patients with moderate to severe mucositis. Benzydamine’s well known anesthetic and anti-inflammatory properties might have contributed to reducing pain and use of opioids, which is a potential factor influencing patients’ compliance with RT. In addition, almost none of the patients in the current study required hospitalization for OM or an associated complication, possibly suggesting that benzydamine could improve healthcare resource utilization relative to what is reported in the literature.

For the future, randomized controlled trials with larger numbers of patients may be useful to control for factors such as age, oral health status/pre-RT dental clearance, oral hygiene regimens during RT, concomitant infections, and smoking.

## Data availability statement

The raw data supporting the conclusions of this article will be made available by the authors, without undue reservation.

## Ethics statement

The study was approved by all relevant regulatory authorities and ethics committees for all clinical trial documentation (protocol 030(Z)WO19247) and adequacy of investigational sites. The study was conducted in accordance with the local legislation and institutional requirements. The participants provided their written informed consent to participate in this study.

## Author contributions

PB: Writing – original draft, Writing – review & editing. VT: Writing – original draft, Writing – review & editing. GDL: Formal Analysis, Writing – original draft, Writing – review & editing. SF: Writing – original draft, Writing – review & editing. ES: Writing – original draft, Writing – review & editing. AC: Writing – original draft, Writing – review & editing.
